# Maternal Vitamin B_12_ Deficiency Detected by Newborn Screening—Evaluation of Causes and Characteristics

**DOI:** 10.3390/nu14183767

**Published:** 2022-09-13

**Authors:** Anna T. Reischl-Hajiabadi, Sven F. Garbade, Patrik Feyh, Karl Heinz Weiss, Ulrike Mütze, Stefan Kölker, Georg F. Hoffmann, Gwendolyn Gramer

**Affiliations:** 1 Division of Neuropediatrics and Metabolic Medicine, Center for Pediatric and Adolescent Medicine, Heidelberg University Hospital, Im Neuenheimer Feld 430, 69120 Heidelberg, Germany; 2 Department of Internal Medicine IV, Gastroenterology and Hepatology, Heidelberg University Hospital, Im Neuenheimer Feld 410, 69120 Heidelberg, Germany; 3 University Medical Center Hamburg-Eppendorf, University Children’s Hospital, Martinistraße 52, 20246 Hamburg, Germany

**Keywords:** maternal vitamin B_12_ deficiency, newborn screening, vitamin supplementation, pregnancy

## Abstract

Vitamin B_12_ deficiency, mostly of maternal origin in newborns, is a well-treatable condition but can cause severe neurologic sequelae in infants. Early detection of vitamin B_12_ deficiency allows the pre-symptomatic treatment of affected children. This evaluation assesses the characteristics of maternal vitamin B_12_ deficiency detected by newborn screening. In a prospective single-center study, a systematic screening strategy for vitamin B_12_ deficiency using a combination of two second-tier strategies was applied. In addition to confirmatory diagnostics in children, the systematic work-up of vitamin B_12_ status was also performed for their mothers. Maternal characteristics were assessed including ethnic origin, diet, and vitamin supplementation during pregnancy. For affected mothers, a work-up by internal medicine was recommended. In total, 121 mother–infant couples were analyzed. 66% of mothers adhered to a balanced diet including meat. The cause of maternal vitamin B_12_ deficiency was unknown in 56% of cases, followed by dietary causes in 32%, and organic causes in 8%. All mothers following a vegan diet and most mothers with a vegetarian diet took vitamin preparations during pregnancy, whereas only 55.8% of mothers with a balanced diet took folic acid or other vitamins. Maternal vitamin B_12_, folic acid, and homocysteine levels were significantly correlated with the child’s folic acid levels, and with homocysteine, methylmalonic, and methylcitric acid levels in first and second NBS dried blood spots. Most children had normal blood counts and showed normocytosis. Although 36.7% of mothers showed anemia, only one presented with macrocytosis. Adherence to vitamin supplementation in pregnancy is low despite the recommendation for supplementation of folic acid. Ideally, the evaluation of mothers for vitamin B_12_ levels and appropriate therapy should be initiated in early pregnancy. In infants detected through newborn screening, the multidisciplinary assessment and therapy of both children and mothers should be performed.

## 1. Introduction

### 1.1. Uptake and Function of Vitamin B_12_

Vitamin B_12_ (cobalamin) is a water-soluble vitamin that is synthesized by microorganisms. Major sources of dietary vitamin B_12_ are foods of animal origin, including meat, milk, eggs, and fish, with plants and fungi contributing little vitamin B_12_ to the human diet [1]. The human body is not able to produce vitamin B_12_ and is therefore dependent on external supply [2]. After dietary intake, vitamin B_12_ underlies a complex pathway of intestinal absorption and intracellular processing. Uptake in the gastrointestinal tract depends on an intrinsic factor, which is synthesized by the gastric parietal cells, and on the cubulin-amnionless (cubam) receptor in the distal ileum [1,2,3,4]. In blood, vitamin B_12_ is bound to transcobalamin or haptocorrin, whereby only transcobalamin-bound vitamin B_12_ (holoTC) is active and available for uptake by CD320, the B_12_ receptor expressed on most cells [1]. Cobalamin functions as a cofactor for two enzymes in human metabolism: methionine synthase and methylmalonyl–coenzyme A mutase [2]. Metabolic changes due to vitamin B_12_ deficiency result from the dysfunction of these enzymes, leading to an increase in the functional parameters homocysteine (Hcy), methylmalonic acid (MMA), and potentially methylcitric acid (MCA) [2,3]. Vitamin B_12_ plays an essential role in all human cells and many organ systems, especially in tissues with a high cell turnover [2]. Along with folic acid, vitamin B_12_ is necessary for the synthesis of deoxyribonucleic acid (DNA) and ribonucleic acid (RNA), lipids, and protein, which are essential for the development, myelination, and normal function of the central and peripheral nervous system. Furthermore, it is required for effective erythropoiesis [2].

### 1.2. Causes and Symptoms of Vitamin B_12_ Deficiency

Inadequate intake, inadequate bioavailability, or malabsorption are reasons for vitamin B_12_ deficiency [5]. The most common cause in adults with a prevalence of 50 to 4000 cases per 100,000 persons is a loss of intrinsic factor due to autoimmune atrophic gastritis (pernicious anemia) [2,4]. Between 2.5% and 26% of the general population is affected by vitamin B_12_ deficiency [5,6,7], with a wide spectrum of disease, from asymptomatic to life-threatening pancytopenia or myelopathy [2]. In women of child-bearing age and pregnant women, vitamin B_12_ deficiency is prevalent with frequencies between 10% and 50% across the world, with especially high rates in studies from the Indian subcontinent, Eastern Mediterranean, and South American regions [2,8,9,10,11,12,13,14]. During pregnancy, low circulating levels of vitamin B_12_ or folic acid have been associated with complications such as neural tube defects, spontaneous abortion, premature birth, and possibly low birth weight [8,15]. Vitamin B_12_ deficiency in newborns is usually of unrecognized maternal origin [11]. Cobalamin status in the neonate depends on maternal cobalamin status during pregnancy, placental function, and gestational age at birth [16]. Newborns affected by vitamin B_12_ deficiency are usually asymptomatic at birth [2,10]. Clinical signs of vitamin B_12_ deficiency appear mostly at the age of 4–6 months [2,11]. Symptoms include developmental delay, muscular hypotonia, irritability, regression, feeding difficulties, and failure to thrive, as well as tremors and seizures [2,11]. Hematologic abnormalities with anemia may be present [2,11]. Untreated vitamin B_12_ deficiency can lead to coma or even death [11]. Because of the late onset of often non-specific symptoms, the diagnosis is often not established until the second half of the first year of life [11,17,18,19]. Many children with symptomatic vitamin B_12_ deficiency show improvement in clinical symptoms after supplementation with vitamin B_12_ [11,17,18,19]. However, long-term neurological and intellectual outcomes following severe and especially long-term vitamin B_12_ deficiency are poor with permanent deficits [11,17,18,19].

### 1.3. Early Detection through Newborn Screening for Vitamin B_12_ Deficiency

Children with vitamin B_12_ deficiency are asymptomatic at birth but may develop severe multisystemic symptoms. As vitamin B_12_ deficiency is a well-treatable condition if identified early, it is a potential target for newborn screening (NBS) [11]. The prevalence of vitamin B_12_ deficiency detected by NBS varies between countries, also depending on the NBS strategies applied. The NBS for vitamin B_12_ deficiency has been shown to be feasible under the application of second-tier strategies with a prevalence between 1 in 30,000 [20] and 1 in 5300 detected newborns in Germany [21,22]. An Italian study found a prevalence of 1 per 5000 in a cohort of 35,000 newborns [12]. The screening program in Estonia reported an incidence of vitamin B_12_ deficiency of 1 in 2500 newborns detected by elevated propionylcarnitine (C3) as an incidental finding in the NBS for methylmalonic and propionic acidemias [23]. The NBS program in the US state of Minnesota reported a detection rate of vitamin B_12_ deficiency in 1 in 33,000 newborns [24].

## 2. Material and Methods

### 2.1. Study Population and Study Design

The study population included 121 pairs of newborns and their mothers. In total, 120 patients were identified in the pilot project Newborn Screening 2020 (NBS 2020/NBS 2025; DRKS-ID DRKS00025324) at the Dietmar Hopp Metabolic Center, Heidelberg University Hospital, between 1 August 2016, and 31 December 2020, with a diagnosis of vitamin B_12_ deficiency or maternal vitamin B_12_ deficiency. One infant born in January 2021 was additionally included because an older sibling had already been diagnosed with vitamin B_12_ deficiency as part of the pilot project.

NBS 2020/NBS 2025 is a prospective single-center study evaluating an extension of the German NBS panel (14 disorders at study initiation, currently 19 target disorders) by an additional 28 conditions [25]. Details on the screening algorithms of NBS for vitamin B_12_ deficiency developed for this study using a combination of two second-tier strategies have been previously published [22]. Primary screening measures used in the second-tier algorithms were C3, ratio C3/acetyl carnitine (C3/C2), methionine (Met), and the ratio of methionine/phenylalanine (Met/Phe). One second-tier strategy analyzed MMA, 3-OH-propionic acid (3-OH-PA), and MCA [26] based on abnormal first-tier results for C3 and C3/C2 (C3 + C3/C2 > cut-off, or C3/C2 > cutoff, or C3 > alarm limit). The second method analyzed total homocysteine (tHcy) [27] after an abnormal first-tier result for Met (<cut-off low) or Met/Phe (<cut-off low or > cut-off high). Patients with vitamin B_12_ deficiency could be reliably identified by elevated tHcy, elevated MMA/MCA, or a combination of both. A diagnosis of “vitamin B_12_ deficiency” was established in cases with elevation of 1 or more functional markers of vitamin B_12_ deficiency (MMA in plasma and/or urine and homocysteine in plasma), in the presence of low vitamin B_12_ serum levels, and of “functional vitamin B_12_ deficiency” in cases with elevation of 1 or more functional markers of vitamin B_12_ deficiency and vitamin B_12_ in the low normal range [28]. Normalization of functional markers and of vitamin B_12_ serum levels under supplementation had to be documented for final confirmation of the diagnosis in the child.

The study NBS 2020 has been approved by the ethics committee of the University Hospital Heidelberg (Number S-533/2015). Written informed consent was obtained from a legal guardian before participation in the study.

### 2.2. Data Collection

Data on the NBS results including second-tier markers as well as confirmatory diagnostics including vitamin B_12_ status and functional markers were collected at the NBS center and metabolic laboratory at Heidelberg University. Data on gestational age, birth weight, and congenital heart defects were obtained from the submission forms, the physicians’ letters, or subsequently by contacting the attending pediatricians. In all cases, a work-up of the maternal vitamin B_12_ status, including functional markers, was recommended, and a laboratory work-up was offered at Heidelberg University Hospital metabolic laboratory in cooperation with the central laboratory in the context of the newborns’ confirmatory diagnostics. In cases with confirmatory diagnostics in the child and/or mother performed in external laboratories, data were retrieved from the transmitted reports. “Maternal vitamin B_12_ deficiency” was diagnosed if the mother’s vitamin B_12_ level was below the normal range and/or functional markers were elevated. A further diagnostic work-up was recommended to mothers with vitamin B_12_ deficiency not clearly explained by dietary reasons and was offered and performed in collaboration with the department of Internal Medicine at the Heidelberg University Hospital. If this work-up was performed at an external hospital, data were retrieved from the submitted reports. Information on mothers’ ethnic origin, diet during pregnancy, vitamin supplementation, cause of vitamin B_12_ deficiency, or subsequent pregnancies was obtained at contact with the family during the work-up of the child or mother or by contacting the mothers or attending pediatricians of the affected newborns. The mothers’ ethnic origin was classified according to topographical criteria as defined by the United Nations Group of Experts on Geographical Names [29].

### 2.3. Study Objective

The primary objective of this analysis was to evaluate causes and characteristics of maternal vitamin B_12_ deficiency detected by abnormal newborn screening of the child and influences on maternal vitamin B_12_ status. Secondary endpoints were the correlation of maternal and infant vitamin B_12_ status and blood counts. Data were also collected on gestational age, birth weight, and congenital heart defects. Furthermore, as part of confirmatory diagnostics concerning vitamin B_12_ deficiency, the initial treatment response of the newborns with vitamin B_12_ deficiency was assessed.

### 2.4. Statistical Analysis

A statistical analysis was performed using IBM SPSS^®^ 28.0. For continuous variables, the mean, median, standard deviation, and interquartile range were calculated. For categorical variables, the absolute and relative numbers were calculated. Correlations of categorical variables were analyzed by cross-tabulations, and statistical significance was determined by a chi-square test with a *p*-value < 0.05. Statistical significance for interval scaled variables was evaluated with a linear regression analysis or multiple linear regression analysis with a *p*-value < 0.05. Statistical significance for dichotomous variables was determined with a logistic regression analysis with a *p*-value < 0.05. Missing data which could not be retrieved and implausible data that could not be verified were treated as cases that were missing for the respective analysis.

## 3. Results

### 3.1. Maternal and Child Characteristics

In total, 121 mother–infant couples were analyzed. Furthermore, 66% of mothers adhered to a balanced diet including meat, followed by rarely meat in 17% of cases, and a vegetarian diet in 9% of cases. 63.3% of mothers took vitamin preparations during pregnancy, and 16 mothers reported feeding disorders. The assumed cause of maternal vitamin B_12_ deficiency was unknown in 56% of cases, followed by dietary causes in 32%, and organic causes in 8%. 43.6% of the mothers had a Central European origin, 30.7% of the mothers were of West Asian origin, and 9.9% were of Southeast European origin.

Infants were born with a mean birth weight of 3138 g ± 583 SD (range 1100–4850 g) at a mean gestational age of 38 weeks ± 2 SD (range 29–42 weeks). In addition, 62.2% of newborns were breastfed, followed by 19.8% who were breastfed and formula-fed, and 16.2% who were formula-fed only. 6.5% of newborns had a congenital heart defect.

Maternal and child characteristics are displayed in Tables S1 and S2 in the Supplementary Materials section.

### 3.2. Maternal Diagnoses

Maternal causes for vitamin B_12_ deficiency were divided into four diagnostic categories: nutritional, organic, nutritional and organic, and unknown cause. In 11 mothers, a comprehensive evaluation was performed in cooperation with physicians from the Department of Internal Medicine at Heidelberg University Hospital. In two cases, the diagnosis of autoimmune gastritis and pernicious anemia with antibodies against parietal cells could be confirmed. Gastritis was suspected in five additional cases, but esophagogastroduodenoscopy was pending at the time of data collection. In three mothers, gastrointestinal malabsorption could be ruled out or diet was identified as the cause of vitamin B_12_ deficiency. Additional diagnoses in the mothers, whose diagnostics were performed internally or externally, included ulcerative colitis, gastric bypass, HELLP (hemolysis, elevated liver enzymes, low platelet count) syndrome, gestational diabetes, severe pancytopenia due to vitamin B_12_ and folic acid deficiency, and carbamazepine treatment during pregnancy. Maternal diagnoses according to the above-named diagnostic categories and vitamin supplementation during pregnancy were not significantly related. Diagnoses and affected subsequent pregnancies were also not significantly correlated. Three out of three mothers (100%) on a vegan diet whose vitamin intake data are known took vitamins during pregnancy. For vegetarian mothers, the percentage was 80% (4 out of 5 mothers). Among mothers who had a balanced diet, 55.8% (24 out of 43 mothers) took vitamins during pregnancy. No significant correlation was found between diet and vitamin intake or type of vitamin supplementation during pregnancy. Similarly, no significant effect was found between diet and iron supplementation. Diet during pregnancy and ethnicity showed no significant correlation. Forty-one mothers were of German origin, and sixty mothers (49.6%) had a migration background. Among two German mothers with a subsequent pregnancy, the siblings were not affected by vitamin B_12_ deficiency. Among the mothers with a migration background, 62.5% (*n* = 5) were not affected, 25% (*n* = 2) subsequent pregnancies suffered again from vitamin B_12_ deficiency, and *n* = 1 remained unknown. Migration background and affected subsequent pregnancies were not significantly correlated.

### 3.3. Maternal Vitamin B_12_ Status

Maternal vitamin B_12_ status was assessed at a mean of 4.5 weeks ± 3.5 SD (range 0–20 weeks) postpartum. Laboratory results are depicted in Table 1. In all, 41.1% (*n* = 44) of mothers had serum vitamin B_12_ levels below the normal range (160–679 pmol/L), with the lowest level being 36 pmol/L.

The influence of diet, cause of maternal vitamin B_12_ deficiency, vitamin supplementation during pregnancy, and ethnic origin on maternal vitamin B_12_ status were analyzed. Neither diet during pregnancy, nor diagnoses, nor vitamin supplementation, nor maternal ethnic origin had a significant effect on serum vitamin B_12_ levels. In addition, no effect on the homocysteine level in plasma could be shown. However, a multiple regression analysis showed that diet, diagnoses, vitamin supplementation, and ethnic origin had an influence on folic acid levels (*p* = 0.038). Mothers with an ethnic origin from West Asia had lower serum folic acid levels by 11.35 units compared to the origin from Middle Europe (*p* = 0.019). There was also an effect on methylmalonic acid levels (conventional method) in urine (*p* = 0.002). Mothers with organic causes of vitamin B_12_ deficiency showed higher values by 12.89 units compared to those with an unknown diagnosis (*p* = 0.009). When considering children’s nutrition and maternal vitamin B_12_ status, an association could be shown with folic acid levels (*p* = 0.048). Mothers whose children were fed by formula or parenteral nutrition showed higher folic acid levels by 25.96 units than mothers whose children were breastfed (*p* = 0.044).

### 3.4. Blood Counts of Mothers and Children

The hemoglobin level and mean corpuscular volume were obtained from mothers and children. The hemoglobin value was classified as normal, anemia, or polyglobuly, and the mean corpuscular volume (MCV) was classified as normo-, micro-, or macrocystosis. The results are depicted in Table 2.

Most children had normal blood counts and normocytosis. Although 36.7% of mothers showed anemia, only one had a blood count with macrocytosis. Diagnosis, diet during pregnancy, and vitamin supplementation were not significantly related to maternal hemoglobin or MCV. However, there was a significant correlation between the type of vitamin intake and hemoglobin (*p* = 0.026), but not with MCV. Mothers who supplemented folic acid during pregnancy were significantly more likely to have normal hemoglobin levels. Iron supplementation had no correlation with the classification of hemoglobin and MCV. A logistic regression analysis showed no effect of maternal vitamin B_12_ status on blood counts. The blood count characteristics of mothers and children were not significantly correlated.

### 3.5. Neonatal Vitamin B_12_ Status

The results of newborns’ vitamin B_12_ status are depicted in Table 3.

### 3.6. Effect of Maternal Vitamin B_12_ Status on Infant Metabolism

The effect of maternal vitamin B_12_ status was analyzed separately on the child’s vitamin B_12_ status for confirmatory diagnostics and screening data.

Maternal vitamin B_12_, folic acid, and homocysteine levels did not show a significant effect on infant vitamin B_12_ levels, neither in the combined, nor in the separate analysis. Maternal MMA and MMA-I levels in plasma and urine also did not have a significant correlation with newborns’ vitamin B_12_ levels. The significant correlations of maternal vitamin B_12_ status on infant metabolism in confirmatory diagnostics are depicted in Table 4.

**Figure 1:**
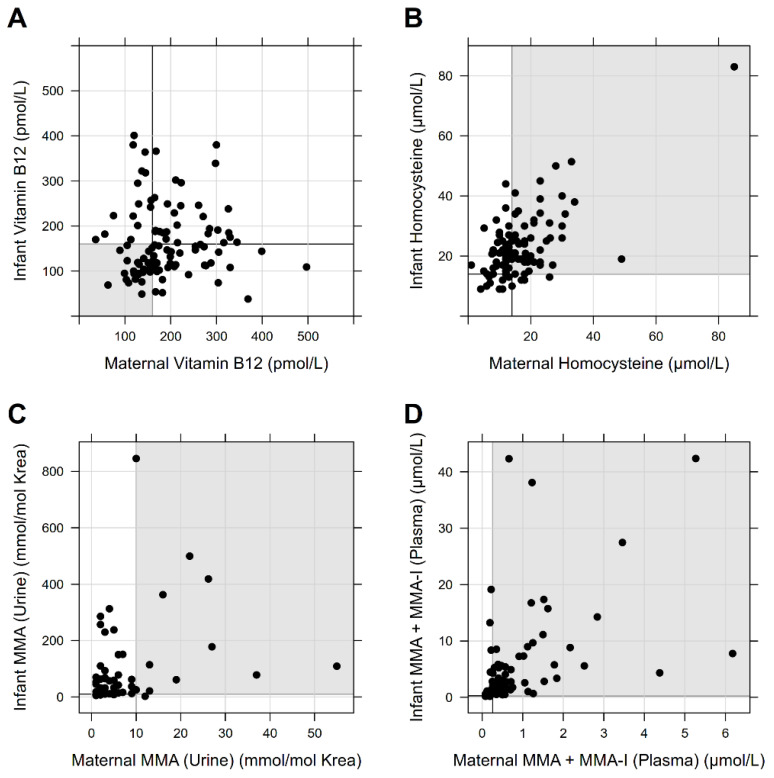
Relation of maternal and infant vitamin B_12_ status. (**A**–**D**) illustrates the correlation of the maternal and infant vitamin B_12_ status based on the vitamin B_12_ levels and the functional parameters Hcy, MMA in urine, and MMA or MMA-I in plasma as a point cloud. Bold lines mark the cut-off of the normal range. Individuals in the gray marked area showed values outside the normal range for both mother and child.

Figure 1 shows the relation of maternal and infant vitamin B_12_ status based on vitamin B_12_ levels and functional parameters.

For NBS data, only the results of second-tier analyses (tHcy, MMA, MCA) were considered. Maternal vitamin B_12_, folic acid, and homocysteine levels showed an impact on the infant homocysteine level in the first NBS sample (*p* < 0.001). The significant correlations of maternal vitamin B_12_ status on the results of the first DBS are depicted in Table 4.

Considering the results of the second-tier analyses in the second DBS, the maternal vitamin B_12_ level showed no effect on tHcy and MCA but on MMA. As in the first DBS, the combined analysis of maternal vitamin B_12_, folic acid, and Hcy also showed a significant effect (*p* < 0.001) on infant tHcy, MMA, and MCA levels from the second DBS. The results are depicted in Table 4.

### 3.7. Effect of Neonatal Nutrition on Newborns’ Vitamin B_12_ Status

The mean value of the serum vitamin B_12_ level at confirmatory diagnostics in exclusively breastfed children was 129 pmol/L with a range of 38 to 249 pmol/L. In children who were not exclusively breastfed but received formula or parenteral nutrition, the mean value was 217 pmol/L with a range of 94 to 401 pmol/L. Neonatal nutrition showed a significant impact on newborns’ vitamin B_12_ status in confirmatory diagnostics but not on the screening data. Neonates who were partially formula fed, formula fed alone, or received a combination of parenteral and formula feeding showed higher serum vitamin B_12_ concentrations than fully breastfed neonates (*p* < 0.001). Infants who were formula fed had significantly lower homocysteine concentrations than newborns who were fully breastfed (*p* = 0.009). There was no significant effect of neonatal nutrition on MMA levels.

### 3.8. Gestational Age, Birth Weight, and Congenital Heart Defects

Maternal vitamin B_12_, Hcy, and MMA levels did not show a correlation with gestational age and birth weight. There was a significant correlation between infants’ vitamin B_12_ levels and gestational age, but not for Hcy and MMA levels. The univariate regression analysis for newborns’ vitamin B_12_ levels showed an inverse effect, the decrease in gestational age in weeks by 0.006 showed an increase of vitamin B_12_ levels by one unit (*p* = 0.012). There was no significant correlation for children’s vitamin B_12_, Hcy, and MMA levels and infants’ birth weight. Three children in this cohort suffered from a congenital heart defect. One child had a ventricular septal defect (VSD), one child suffered from a patent foramen ovale (PFO), and one child had a PFO and a patent ductus arteriosus (PDA). The child with the PDA was born at 29 weeks of gestation.

### 3.9. Infants’ Response to Therapy

**Figure 2:**
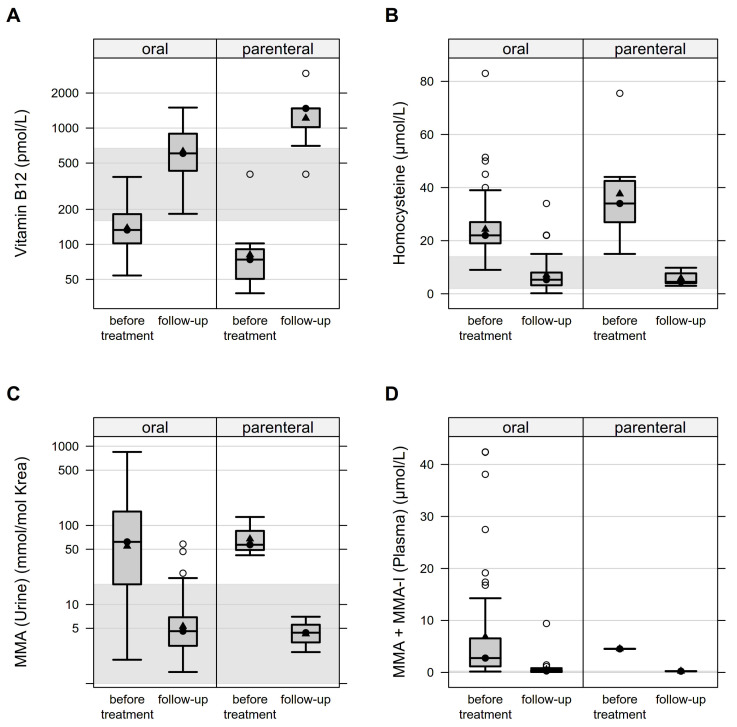
Therapeutic response of vitamin B_12_ supplementation: oral versus parenteral administration. Figure 2 (**A**–**D**) represents the infants’ response to therapy with oral versus also parenteral vitamin supplementation as a boxplot. The gray marked area represents the normal value range. There is an increase in the vitamin B_12_ level and a decrease in the functional parameters Hcy, MMA in urine, and MMA or MMA-I in plasma.

The confirmation of vitamin B_12_ deficiency requires the evaluation of the initial response to treatment. One hundred and ten infants received treatment with vitamin B_12_. In six cases in which only the mother was affected by vitamin B_12_ deficiency, no therapy was performed for the child. Four of those children received formula feeding exclusively, one child was breast and formula fed, and one child received parenteral and formula feeding. In the remaining 5 children with vitamin B_12_ deficiency who did not receive therapy, 4 children were fed only by formula and 1 child was breastfed and formula fed. Of the 108 newborns whose supplementation scheme was known, 99 (91.7%) received oral, *n* = 2 (1.9%) intramuscular, *n* = 5 (4.6%) a combination of oral and intramuscular, *n* = 1 (0.9%) intravenous, and *n* = 1 (0.9%) a combination of intravenous and oral therapy. Treatment was started at a mean age of 4 weeks (SD = 3.1) and first follow-up samples were analyzed at a mean age of 7 weeks (SD = 3.6). Comparisons with oral-only versus also parenteral therapy showed mean percent increases in vitamin B_12_ levels of 78% and 1254%, respectively, with parenteral therapy resulting in vitamin B_12_ concentrations well above the normal range with a mean of 1422 pmol/L (SD = 807.5) at follow-up. Homocysteine levels showed a mean percent decrease of 70% with oral-only versus 80% with parenteral therapy. Plasma or urine MMA concentrations (conventional method and isotopes) showed mean percentage decreases of 90–92% with oral-only and 92–96% with parenteral treatment. Figure 2 illustrates the therapeutic response of purely oral versus also parenteral supplementation based on the increase in vitamin B_12_ levels and decrease in functional parameters.

## 4. Discussion

A diagnostic work-up of vitamin B_12_ deficiency in newborns should always include a comprehensive analysis of the maternal vitamin B_12_ status and diet, as vitamin B_12_ deficiency in children of this age group is mostly of maternal origin. Here, we report the results of the confirmatory work-up in mothers of 121 children with vitamin B_12_ deficiency detected by an NBS pilot study.

### 4.1. Maternal Diet and Causes of Vitamin B_12_ Deficiency

In this study, about two-thirds of mothers with vitamin B_12_ deficiency reported a balanced diet including meat. A vegan or vegetarian diet, as described frequently in other screening programs [22,30,31], was rarely reported in this cohort. Recommendations from the German Federal Center for Nutrition include a balanced diet and the initiation of folate supplementation before pregnancy for all women [22,32]. For women who follow a vegetarian or vegan diet, the initiation of micronutrient supplementation before pregnancy, including vitamin B_12_, and monitoring throughout pregnancy is recommended [22,32]. Despite a general recommendation for folic acid supplementation in Germany [32], only 63% of women in our cohort had taken any vitamin supplementation during pregnancy. This low compliance with the recommendations on prenatal folic acid supplementation is consistent with previous national surveys in Germany [11,33]. In other countries, adherence to prenatal B-vitamin supplementation is much higher, e.g., 93% in a Canadian study [11,34]. Some mothers reported feeding difficulties in pregnancy, iron supplementation due to anemia, or use of carbamazepine during pregnancy. Despite these risk factors [35] or symptoms, no diagnostic work-up for vitamin B_12_ deficiency had been performed during pregnancy. This emphasizes that caregivers of pregnant women should pay increased attention to vitamin B_12_ deficiency. In women with anemia, the coexistence of iron, folate, and vitamin B_12_ deficiency has been reported [22,36]. Hematologic changes caused by vitamin B_12_ deficiency may be masked by concomitant iron deficiency [22,36]. This is also reflected in the blood counts of the mothers in this cohort, who mostly showed normocytic or microcytic anemia. Therefore, the isolated evaluation of blood counts in pregnancy is not sufficient to differentiate maternal vitamin B_12_ deficiency from other nutritional deficiencies. Even in the presence of iron deficiency, vitamin B_12_ should be determined in anemic women. National German maternity guidelines do currently not include the routine evaluation of vitamin B_12_ status [22,32]. As women with gastrointestinal malabsorption would not benefit from a general recommendation for vitamin B_12_ supplementation in pregnancy and are often clinically asymptomatic, an ideal prevention strategy would include the routine testing of vitamin B_12_ status in early pregnancy [11,30]. Vitamin B_12_ deficiency can also be associated with HELLP syndrome [37] or gestational diabetes [38], which were also present in our cohort and again have implications for the fetus.

In the majority of mothers from our cohort, no gastrointestinal cause for vitamin B_12_ deficiency was found. Most mothers in this cohort had an unknown reason for vitamin B_12_ deficiency followed by dietary causes. Although further diagnostics were recommended for all mothers without a clear nutritional cause of vitamin B_12_ deficiency, a thorough etiological work-up by internal medicine was only performed in a minority of cases. This work-up was able to clarify the reasons in 90% of cases. This emphasizes the advantages of a structured work-up in parallel to confirmatory diagnostics in the child in cooperation with internal medicine in maternal vitamin B_12_ deficiency detected by NBS. Mothers in our study were of various ethnic origins, and 50% had a migration background. This is an overrepresentation of women with a migration background compared to the general population in Germany of about 27% [29]. The lack of vitamin supplementation during pregnancy may particularly affect women with a migration background due to a lack of information, language barriers, or greater hesitation to attend preventive prenatal care appointments [11,22,39]. However, in this cohort, ethnicity showed no significant association with diet, vitamin supplementation, or subsequent affected pregnancies. Nevertheless, it must be taken into account that, in contrast to other evaluations [21], ethnic background was considered in the analysis, but not language skills or school education which might also play a role.

In two cases, subsequent pregnancies were again affected by vitamin B_12_ deficiency despite the diagnosis being previously established in the older sibling and mother. Considering the high rate of unknown maternal causes as mentioned above and the fact that there has been only little systematic etiologic evaluation by internal medicine apart from the cooperation with the Department of Internal Medicine at the Heidelberg study site, a collaborative evaluation program for affected children with vitamin B_12_ deficiency and their mothers is recommended for an optimal prevention strategy.

### 4.2. Maternal and Infants’ Vitamin B_12_ Status

A meta-analysis showed that rates of vitamin B_12_ insufficiency are especially high in certain populations, e.g., the Indian subcontinent and Eastern Mediterranean [13]. In our cohort, however, there was no effect of ethnic background on maternal vitamin B_12_ status, but rather on folic acid levels. As the cause of vitamin B_12_ deficiency, diet during pregnancy, and vitamin supplementation did not correlate with migration background, the cause of this effect remains unclear. More than half of the mothers had a normal vitamin B_12_ status at confirmatory evaluation, and maternal vitamin B_12_ status showed no significant correlation with infant vitamin B_12_ status. This could be explained by the fact that laboratory values of mothers were taken on average 5 weeks postpartum. In a study from Norway, maternal vitamin B_12_ levels were shown to increase again about 6 weeks after birth [16]. The postpartum increase in maternal serum cobalamin may be a physiologic adaptation to enhance the cobalamin transfer to the infant by increasing the cobalamin concentration in breast milk [16]. Maternal vitamin B_12_ concentration at the gestational age of 18 weeks has been shown to be a predictor of cobalamin, tHcy, and MMA concentrations in the mother during pregnancy and lactation and in the infant at 6 months of age [16].

It cannot be excluded that some children identified with milder functional vitamin B_12_ deficiency would not have developed symptoms without treatment. This depends on the choice of nutrition following birth. Breast feeding, which was the preferred way of child nutrition in our study, by a mother with vitamin B_12_ deficiency or suboptimal vitamin B_12_ status poses a relevant risk, as reported cases diagnosed with severe clinical symptoms of vitamin B_12_ deficiency had vitamin B_12_ levels at diagnosis of about 100 pmol/L after prolonged episodes of exclusive breast milk feeding. These cases might well have had higher neonatal vitamin B_12_ levels still resulting in symptomatic vitamin B_12_ deficiency with breast feeding by a vitamin B_12_-deficient mother [40].

A serum vitamin B_12_ level of >394 pmol/L at week 18 of pregnancy is recommended [16]. Therefore, vitamin B_12_ levels of pregnant women need to be carefully assessed early in pregnancy to provide appropriate recommendations for diet and supplementation during pregnancy and to ensure an optimal fetal and neonatal nutrient supply [16,41].

### 4.3. Gestational Age, Birth Weight, and Congenital Heart Defects

In a meta-analysis from Norway [42], maternal vitamin B_12_ deficiency was associated with preterm birth. This correlation could not be shown in our evaluation. Data on the relationship between vitamin B_12_ levels and birth weight have been controversial [41]. Vitamin B_12_ deficiency has been associated with low birth weight and intrauterine growth restriction [41,42]. However, some studies did not find any association [41,43,44,45]. In our cohort, neither infant vitamin B_12_ levels nor maternal vitamin B_12_ levels showed any association with gestational age or birth weight. However, it must be emphasized that in our analysis, maternal levels were determined postpartum, whereas in the study from India [41], low birth weight was associated with low maternal vitamin B_12_ levels in the first and second trimesters.

Other studies have postulated an association between vitamin B_12_ deficiency or hyperhomocysteinemia and congenital heart defects [46]. The birth prevalence of congenital heart defects is 9 per 1000 live births [47,48]. In our cohort, one patient suffered from a VSD resulting in a prevalence of 2.1% with respect to all cases with data reported on this aspect. However, in relation to the total cohort of 121 cases, the prevalence is only 0.08%, which is in accordance with the overall prevalence in the normal population. Thus, given the limitation of available data, no clearly increased incidence of congenital heart defects could be shown for newborns in this cohort.

### 4.4. Infantiles’ Response to Therapy

Most children were treated by an oral supplementation regimen developed for this NBS pilot study. The treatment response as part of confirmatory diagnostics and clinical follow-up showed that this is an effective and feasible alternative to invasive intramuscular applications [21,22,49]. The parenteral supplementation regimen (including intramuscular and intravenous supplementation) showed a higher response in terms of vitamin B_12_ levels but may also point to an over-treatment given the supranormal vitamin B_12_ levels achieved. An at least normal range of vitamin B_12_ levels and rapid normalization of functional parameters are reached with both oral and parenteral treatment.

## 5. Conclusions

Vitamin B_12_ deficiency is associated with adverse outcomes for mother and child if untreated. Early detection of maternal vitamin B_12_ deficiency already in pregnancy would be desirable but is complex and currently not sufficiently performed. Maternal blood counts alone do not allow for the differentiation of vitamin B_12_ deficiency and other, possibly combined, nutritional deficiencies. NBS for vitamin B_12_ deficiency is feasible and allows early treatment in children and further work-up and treatment for mothers. After detection of vitamin B_12_ deficiency in NBS, a systematic multidisciplinary evaluation and therapy of both children and mothers should be performed. This is of benefit for both children and mothers and can also prevent affected subsequent pregnancies.

## Figures and Tables

**Table 1:** Maternal vitamin B_12_ status.

		Normal Range
**Vitamin B_12_ (S)** (Valid data: *n* = 107; missing data *n* = 14)		160–679 pmol/L
Mean ± SD	189 ± 79	
Median (IQR)	168 (137; 223)	
Minimum/maximum	36/497	
**Folic Acid (S)** (Valid data: *n* = 97; missing data *n* = 24)		4.5–21 nmol/L
Mean ± SD	18.1 ± 12.7	
Median (IQR)	13.0 (8.0; 23.5)	
Minimum/maximum	5.0/45.0	
**Total Hcy (P)** (Valid data: *n* = 102; missing data: *n* = 19)		2–14 µmol/L
Mean ± SD	16.6 ± 10.2	
Median (IQR)	14.0 (11.0; 20.2)	
Minimum/maximum	1.0/85.0	
MMA + MMA-I (P) ** (Valid data: *n* = 87; missing data: N =34)		<0.26 µmol/L
Mean ± SD	0.85 ± 1.07	
Median (IQR)	0.45 (0.25; 1.05)	
Minimum/maximum	0.08/6.18	
**MMA (U)** (Valid data: *n* = 63; missing data: *n* = 58)		<10 mmol/mol Creatinine
Mean ± SD	7.4 ± 9.5	
Median (IQR)	4.0 (2.0; 9.0)	
Minimum/maximum	1.0/55.0	
MMA-I (U) ** (Valid data: *n* = 83; missing data: *n* = 38)		<18 mmol/mol Creatinine
Mean ± SD	4.9 ± 9.8	
Median (IQR)	2.1 (1.5; 4.7)	
Minimum/maximum	0.4/79.8	

** MMA-I: methylmalonic acid quantification using stable-isotope labeled D3-MMA as internal standard.

**Table 2:** Blood counts of mothers and children.

**Maternal hemoglobin** (Valid data: *n* = 31; missing data: *n* = 90)	
Normal	20 (64.5%)
Anemia	11 (35.5%)
**Maternal MCV** (Valid data: *n* = 31; missing data: *n* = 90)	
Normocytosis	22 (71.0%)
Microcytosis	8 (25.8%)
Macrocytosis	1 (3.2%)
**Newborn hemoglobin** (Valid data: *n* = 45; missing data: *n* = 76)	
Normal	35 (77.8%)
Anemia	7 (15.5%)
Polyglobuly	3 (6.7%)
**Newborn MCV** (Valid data: *n* = 42; missing data: *n* = 79)	
Normocytosis	37 (88.1%)
Microcytosis	2 (4.8%)
Macrocytosis	3 (7.1%)

**Table 3:** Newborns’ vitamin B_12_ status.

Confirmation Data		Normal Range
**Vitamin B_12_ (S)** (Valid data: *n* = 118; missing data *n* = 3)		160–670 pmol/L
Mean ± SD	161 ± 79	
Median (IQR)	144 (102; 191)	
Minimum/maximum	38/401	
**Folic Acid (S)** (Valid data: *n* = 108; missing data *n* = 13)		4.5–21 nmol/L
Mean ± SD	34.6 ± 9.3	
Median (IQR)	36.0 (27.0; 45.0)	
Minimum/maximum	14.0/46.0	
**Hcy (P)** (Valid data: *n* = 117; missing data: *n* = 4)		2–14 µmol/L
Mean ± SD	23.3 ± 11.3	
Median (IQR)	21.0 (17.0; 27.0)	
Minimum/maximum	9.0/83.0	
**MMA + MMA-I (P)** (Valid data: *n* = 85; missing data *n* = 36)		<0.26 µmol/L
Mean ± SD	5.64 ± 8.47	
Median (IQR)	2.60 (0.91; 5.79)	
Minimum/maximum	0.17/42.37	
**MMA (U)** (Valid data: *n* = 86; missing data: *n* = 35)		<18 mmol/mol Creatinine
Mean ± SD	105 ± 145	
Median (IQR)	50 (16; 133)	
Minimum/maximum	2/846	
**MMA-I (U)** (Valid data: *n* = 94; missing data: *n* = 27)		<10 mmol/mol Creatinine
Mean ± SD	99.9 ± 190.0	
Median (IQR)	38.5 (14.4; 100.4)	
Minimum/maximum	2.3/1368.8	
**Screening Data**		**Normal Range**
**Hcy (DBS 1)** (*n* = 116)		<12 (P99.2) µmol/L
Mean ± SD	17.1 ± 7.3	
Median (IQR)	15.9 (12.9; 20.4)	
Minimum/maximum	2.9/50.5	
**MMA (DBS 1)** (*n* = 80)		2.14 (P99.9), 2.35 (P99.9) *** µmol/L
Mean ± SD	5.55 ± 10.47	
Median (IQR)	2.30 (1.69; 6.15)	
Minimum/maximum	0.06/85.71	
**MCA (DBS 1)** (*n* = 90)		0.08 (P99.9)/0.34 (P99.9) *** µmol/L
Mean ± SD	0.35 ± 0.58	
Median (IQR)	0.18 (0.10; 0.37)	
Minimum/maximum	0/4.62	
**Hcy (DBS 2)** (*n* = 107)		<12 (P99.2) µmol/L
Mean ± SD	15.6 ± 6.9	
Median (IQR)	14.7 (10.0; 20.4)	
Minimum/maximum	4.9/46.0	
**MMA (DBS 2)** (*n* = 94)		2.14 (P99.9), 2.35 (P99.9) *** µmol/L
Mean ± SD	5.10 ± 6.28	
Median (IQR)	2,79 (1.94; 5.89)	
Minimum/maximum	0/34.40	
**MCA (DBS 2)** (*n* = 102)		0.08 (P99.9)/0.34 (P99.9) *** µmol/L
Mean ± SD	0.37 ± 0.46	
Median (IQR)	0.26 (0.16; 0.42)	
Minimum/maximum	0/2.84	

*** During the study the method for second-tier measurements MMA, 3-OH-PA, and MCA was adapted to new reagents; therefore, the cut-off for these parameters changed. P: percentile.

**Table 4:** Effect of maternal vitamin B_12_ status on infant metabolism.

Maternal Vitamin B_12_ Status	Infant Vitamin B_12_ Status	*p*-Value
Confirmation Data		
Folic acid	Folic acid	*p* < 0.001
Increase in maternal folic acid concentration by one unit indicates a 0.2 increase in infant folic acid concentration.		
Homocysteine	Homocysteine	*p* < 0.001
Increase in maternal Hcy concentration by one unit indicates a 0.7 increase in infant Hcy concentration.		
Homocysteine	MMA (U)	*p* = 0.002
Increase in maternal Hcy concentration by one unit indicates a 5.1 increase in infant MMA (U) concentration.		
Homocysteine	MMA-I (U)	*p* < 0.001
Increase in maternal Hcy concentration by one unit indicates a 9.8 increase in infant MMA-I (U) concentration.		
Homocysteine	MMA or MMA-I (P)	*p* = 0.018
Increase in maternal Hcy concentration by one unit indicates a 0.25 increase in infant MMA or MMA-I in plasma concentration.		
MMA + MMA-I (P)	MMA or MMA-I (P)	*p* < 0.001
Increase in maternal MMA and MMA-I in plasma concentration by one unit indicates a 3.66 increase in infant MMA and MMA-I in plasma concentration.		
**NBS data (DBS 1)**		
Homocysteine	Homocysteine	*p* < 0.001
Increase in maternal Hcy concentration by one unit indicates a 0.2 increase in infant Hcy concentration.		
Vitamin B_12_	Homocysteine	*p* = 0.009
Increase in maternal vitamin B_12_ concentration by one unit indicates a 0.02 decrease in infant Hcy concentration.		
MMA + MMA-I (P)	Homocysteine	*p* < 0.001
Increase in maternal MMA and MMA-I in plasma concentration by one unit indicates a 2.78 increase in infant Hcy concentration.		
MMA (U)	Homocysteine	*p* = 0.025
Increase in maternal MMA concentration in urine by one unit indicates a 0.31 increase in infant Hcy concentration.		
Homocysteine	MMA	*p* < 0.001
Increase in maternal Hcy concentration by one unit indicates a 0.8 increase in infant MMA concentration.		
Vitamin B_12_	MMA	*p* = 0.015
Increase in maternal Vitamin B_12_ concentration by one unit indicates a 0.04 decrease in infant MMA concentration.		
MMA + MMA-I (P)	MMA	*p* < 0.001
Increase in maternal MMA and MMA-I in plasma concentration by one unit indicates a 6.44 increase in infant MMA concentration.		
MMA (U)	MMA	*p* = 0.034
Increase in maternal MMA concentration in urine by one unit indicates a 0.7 increase in infant MMA concentration.		
Folic acid	MCA	*p* = 0.014
Increase in maternal folic acid concentration by one unit indicates a 0.1 increase in infant MCA concentration.		
Homocysteine	MCA	*p* < 0.001
Increase in maternal Hcy concentration by one unit indicates a 0.45 increase in infant MCA concentration.		
MMA + MMA-I (P)	MCA	*p* < 0.001
Increase in maternal MMA and MMA-I in plasma concentration by one unit indicates a 0.36 increase in infant MCA concentration.		
**NBS data (DBS 2)**		
Vitamin B_12_	MMA	*p* = 0.02
Increase in maternal vitamin B_12_ concentration by one unit indicates a 0.21 decrease in infant MMA concentration.		
Homocysteine	Homocysteine	*p* < 0.001
Increase in maternal Hcy concentration by one unit indicates a 0.2 increase in infant Hcy concentration.		
Homocysteine	MMA	*p* < 0.001
Increase in maternal Hcy concentration by one unit indicates a 0.32 increase in infant MMA concentration.		
Homocysteine	MCA	*p* < 0.001
Increase in maternal Hcy concentration by one unit indicates a 0.02 increase in infant MCA concentration.		
MMA + MMA-I (P)	Homocysteine	*p* < 0.001
Increase in maternal MMA and MMA-I in plasma concentration by one unit indicates a 0.4 increase in infant Hcy concentration.		
MMA + MMA-I (P)	MMA	*p* < 0.001
Increase in maternal MMA and MMA-I in plasma concentration by one unit indicates a 2.54 increase in infant MMA concentration.		
MMA + MMA-I (P)	MCA	*p* = 0.005
Increase in maternal MMA and MMA-I in plasma concentration by one unit indicates a 0.14 increase in infant MCA concentration.		
MMA (U)	MMA	*p* = 0.033
Increase in maternal MMA in urine concentration by one unit indicates a 0.22 increase in infant MMA concentration.		

## Supplementary Materials

The following are available online at https://www.mdpi.com/article/10.3390/nu14183767/s1, Table S1: Maternal characteristics; Table S2: Child characteristics.
