# Supplementary material for: Maternal Vitamin B12 Deficiency Detected by Newborn Screening—Evaluation of Causes and Characteristics
Source: Nutrients. 2022 Sep 13;14(18):3767. doi: 10.3390/nu14183767 (PMC9505342; doi:10.3390/nu14183767)
Supplement: Supplementary file 1 [file nutrients-14-03767-s001.zip › nutrients-1910674-supplementary.pdf]

Table S1. Maternal characteristics.

**Age at birth of child (years)**<br>(Valid data: N=113; missing data: N=8)	
Mean ± SD	30 ± 5
Median (IQR)	31 (27; 35)
Minimum/maximum	14/49
**Diet during pregnancy***<br>(Valid data: N=100; missing data: N=21)	
Balanced including meat	66 (66.0%)
Rarely meat	17 (17.0%)
Vegetarian	9 (9.0%)
Vegan	5 (5.0%)
Unbalanced	3 (3.0%)
**Vitamin supplementation during pregnancy***<br>(Valid data: N=69; missing data: N=52)	
Yes	43 (63.3%)
No	26 (37.7%)
**Type of vitamin supplementation***<br>(Valid data from N=69: N=43; missing data: N=26)	
Folic acid	22 (51.2%)
Folic acid + vitamin B_{12}	13 (30.2%)
Vitamin B_{12}	2 (4.7%)
Multivitamins	5 (11.6%)
Unknown	1 (2.3%)
**Time of vitamin supplementation***<br>(Valid data from N=69: N=43; missing data: N=26)	
First trimenon	10 (23.3%)
Complete pregnancy	13 (30.2%)
Unknown	20 (46.5%)
**Iron supplementation***<br>(Valid data: N=75; missing data: N=46)	
Yes	25 (33.3%)
No	50 (66.7%)
**Feeding disorders during pregnancy***<br>(N=16 mothers reported feeding disorders)	
Poor eater	4 (25.0%)
Hyperemesis	5 (31.3%)
Aversion against meat	4 (25.0%)
Heartburn	1 (6.3%)
Aversion against meat + hyperemesis	2 (12.5%)
**(Assumed) cause for maternal vitamin B_{12} deficiency***<br>(N=121)	
Diet	39 (32.2%)
Organic cause	10 (8.3%)
Diet + organic cause	4 (3.3%)
Unknown	68 (56.2%)
**Consecutive pregnancy***<br>(Valid data: N=62; missing data: N=59)	
Yes	11 (17.7%)
No	51 (82.3%)
**Following sibling affected***<br>(N=11)	

Yes	2 (18.2%)
No	7 (63.6%)
Unknown	2 (18.2%)
**Ethnic origin***<br>(Valid data: N=101; missing data: N=20)	
Middle Europe (German, Polish, Hungarian, Kroatian)	44 (43.6%)
Eastern Europe (Ukranian, Russian)	4 (4.0%)
Western Europe (Dutch)	1 (1.0%)
Southeastern Europe (Romanian, Albanian, Bulgarian, Macedonian, Kosovan)	10 (9.9%)
Southern Europe (Italian)	1 (1.0%)
West Asia (Georgian, Turkish, Syrian, Iraki)	31 (30.7%)
South Asia (Afghan, Pakistani)	5 (5.0%)
East Asia (Chinese)	1 (1.0%)
East Africa (Egypt, Eritrean, East African)	3 (3.0%)
South America (Brazilian)	1 (1.0%)

*Absolute and relative numbers.

Table S2. Child characteristics.

**Sex*** (N=121)	
Male	64 (52.9%)
Female	57 (47.1%)
**Birth weight (grams)** (N=121)	
Mean ± SD	3138 ± 583
Median (IQR)	3180 (2795; 3500)
Minimum/maximum	1100/4850
**Gestational age (weeks)** (N=121)	
Mean ± SD	38 ± 2
Median (IQR)	39 (38; 40)
Minimum/maximum	29/42
**Nutrition at time of confirmatory diagnostics*** (Valid data: N=111; missing data: N=10)	
Breast milk	69 (62.2%)
Formula feeding	18 (16.2%)
Breast milk + formula feeding	22 (19.8%)
Parenteral nutrition + formula feeding	2 (1.8%)
**Diagnosis*** (N=121)	
Vitamin B_{12} deficiency	115 (95%)
Only maternal vitamin B_{12} deficiency	6 (5%)
**Treatment*** (N=121)	
Yes	110 (90.9%)
No	11 (9.1%)
**Congenital Heart Defect*** (Valid data: N=46; missing data: N=75)	
Yes	3 (6.5%)
No	43 (93.5%)

*Absolute and relative numbers.
